# Acute Systemic Infection with Dengue Virus Leads to Vascular Leakage and Death through Tumor Necrosis Factor-α and Tie2/Angiopoietin Signaling in Mice Lacking Type I and II Interferon Receptors

**DOI:** 10.1371/journal.pone.0148564

**Published:** 2016-02-04

**Authors:** Supranee Phanthanawiboon, Kriengsak Limkittikul, Yusuke Sakai, Nobuyuki Takakura, Masayuki Saijo, Takeshi Kurosu

**Affiliations:** 1 Research Institute for Microbial Diseases, Osaka University, Suita, Osaka, Japan; 2 Department of Tropical Pediatrics, Faculty of Tropical Medicine, Mahidol University, Bangkok, Thailand; 3 Department of Virology I, National Institute of Infectious Diseases, Shinjyuku, Tokyo, Japan; Institut Pasteur of Shanghai, CHINA

## Abstract

Severe dengue is caused by host responses to viral infection, but the pathogenesis remains unknown. This is, in part, due to the lack of suitable animal models. Here, we report a non-mouse-adapted low-passage DENV-3 clinical isolate, DV3P12/08, derived from recently infected patients. DV3P12/08 caused a lethal systemic infection in type I and II IFN receptor KO mice (IFN-α/β/γR KO mice), which have the C57/BL6 background. Infection with DV3P12/08 induced a cytokine storm, resulting in severe vascular leakage (mainly in the liver, kidney and intestine) and organ damage, leading to extensive hemorrhage and rapid death. DV3P12/08 infection triggered the release of large amounts of TNF-α, IL-6, and MCP-1. Treatment with a neutralizing anti-TNF-α antibody (Ab) extended survival and reduced liver damage without affecting virus production. Anti-IL-6 neutralizing Ab partly prolonged mouse survival. The anti-TNF-α Ab suppressed IL-6, MCP-1, and IFN-γ levels, suggesting that the severe response to infection was triggered by TNF-α. High levels of TNF-α mRNA were expressed in the liver and kidneys, but not in the small intestine, of infected mice. Conversely, high levels of IL-6 mRNA were expressed in the intestine. Importantly, treatment with Angiopoietin-1, which is known to stabilize blood vessels, prolonged the survival of DV3P12/08-infected mice. Taken together, the results suggest that an increased level of TNF-α together with concomitant upregulation of Tie2/Angiopoietin signaling have critical roles in severe dengue infection.

## Introduction

Dengue fever is caused by dengue virus (DENV), which is transmitted by mosquitoes. The worldwide incidence of dengue fever has increased markedly in recent decades; indeed, at least 2.5 billion people (approximately 40% of the global population) are now at risk. The World Health Organization estimates that there may be 390 million DENV infections worldwide every year, resulting in approximately 25,000 deaths [1]. At present, no effective vaccines or drugs are available. There are four serotypes of DENV: DENV-1–4, which cause a number of conditions, including undifferentiated fever, dengue fever (DF), dengue hemorrhagic fever (DHF), and dengue shock syndrome (DSS) [2].

DENV belongs to the family *Flaviviridae* within the genus *Flavivirus*. The genus *Flavivirus* comprises arthropod-borne viruses such as yellow fever virus, Japanese encephalitis virus, West Nile virus, and DENV [3]. The DENV genome comprises a single-stranded RNA molecule of 10.7 kb, which encodes a single precursor polyprotein that is co- and post-translationally processed by viral and cellular proteases to yield three structural proteins (the capsid, pre-membrane, and envelope proteins) and seven non-structural proteins (NSs), namely, NS1, NS2A, NS2B, NS3, NS4A, NS4B, and NS5.

The signs of severe dengue virus infection include plasma leakage into interstitial spaces and thrombocytopenia [4, 5], which result in the life-threatening syndrome, DHF. There are two hypotheses to explain the pathogenesis of DHF. One is based on the virulence of the infecting DENV: virulent dengue virus strains cause DHF, while avirulent DENV strains cause DF [5]. The other is based on immunopathogenesis, and suggests that DHF is mediated by host immune responses. Both factors are likely to be intricately associated each other. Most importantly, it is known that severe disease is due to the host response to DENV infection [5]. For this reason, establishing a small-animal model of the disease is necessary if we are to fully understand the interaction between DENV and host response, and its pathogenesis and develop effective anti-dengue therapeutics. The development of a suitable animal model for DENV infection, however, has been hampered by the low (or lack of) viral replication in wild-type mice, even in type I and II IFN receptor-deficient mice (AG129 mice); DENV only replicates in the latter when they are infected with a high dose of mouse-adapted virus [6].

Here, we developed a new mouse model of a lethal DENV-3 infection, which was characterized by DHF-like vascular leakage. Intraperitoneal infection with low-passage DENV-3 P12/08 (DV3P12/08) isolated from infected Thai patient caused an acute systemic disease in C57BL/6 background knockout (KO) mice lacking type I interferon (IFN)-α/β receptors (IFN-α/βR KO mice) and in mice lacking both type I and II IFN receptors (IFN-α/β/γR KO mice). Infection by DV3P12/08 caused vascular leakage in the liver, kidney, and small intestine, and tissue damage in the spleen and liver. High levels of virus production were observed in the spleen, liver, kidney, thymus, lung, peritoneal exudate cells (PEC), bone marrow (BM), intestine, and serum, but not in the brain. High levels of TNF-α, IL-6, and MCP-1 were detected in the serum during the final stages of the disease. TNF-α mRNA was mainly produced in the liver and kidney, while high levels of IL-6 mRNA were produced in the intestine. A neutralizing anti-TNF-α antibody (Ab), suppressed increases in the levels of IL-6, MCP-1 and IFN-γ, protected mice from liver damage, and prolonged mouse survival. Importantly, angiopoietin-1, which is a stabilizer of blood vessels, protected mice from the early lethal effects of DENV. Thus the mouse model described herein may provide new insights into the pathogenesis of DHF.

## Materials and Methods

### Ethical statements

The samples including DV3P12/08 were from an already-existing collection and the study was approved by the ethics committee of Institute for the Development of Human Research Protections, Thailand. All samples were anonymized. All animal experiments were performed in accordance with the guidelines for the care and use of laboratory animals at the Research Institute for Microbial Diseases, Osaka University. The study was approved by the Animal Experiment Committee of the Research Institute for Microbial Diseases, Osaka University (#H25-09-1), as specified in the Fundamental guidelines for the Proper Conduct of Animal Experiment and Related Activities in Academic Research Institutions under the jurisdiction of the Ministry of Education, Culture, Sports, Science and Technology, Japan, 2006. Trained laboratory personnel performed anesthesia of mice via intraperitoneal injection of a mixture of medetomidine, midazolam, and butorphanol during viral injection and euthanasia by cervical dislocation.

### Virus and cells

The virus strain DV3P12/08 was derived from patients infected with DENV-3 in Thailand in 2008, who exhibited DHF grade 1. The virus was isolated in *Aedes albopictus* C6/36 cells and then passaged 3–5 times in C6/36 cells and once in Vero cells. Virus stocks were stored at -80°C until use. The C6/36 cell line was cultured at 28°C in Leibovitz’s 15 medium (Gibco, Gland Island, NY) supplemented with 0.3% BactoTM Tryptose Phosphate Broth (Becton Dickinson, Sparks Glencoe, MD, USA) and 10% fetal calf serum (FCS). Vero cells were cultured in Eagle’s minimum essential medium (Nacalai Tesque, Kyoto, Japan) supplemented with 10% FCS.

### Focus-forming assays

Virus infectivity was measured in focus-forming assays and expressed as focus-forming units (FFU). Briefly, culture supernatants were serially diluted (10-fold) in MEM and then 50 ml of diluent was added to monolayers of Vero cells in 96-well microplates. After incubating for 2 h at 37°C, the infected cells were treated with 2% carboxyl-methyl-cellulose (CMC) in 2% FBS-MEM for a further 72 h. The infected cells were then washed five times, fixed in a 3.7% formaldehyde solution, and permeabilized with 0.1% Triton X-100 prior to overnight incubation with an anti-E monoclonal antibody (D23-1G7C2) [7] at 4°C. The infected cells were then washed and incubated with horseradish peroxidase (HRP)-conjugated rabbit anti-human IgG (309-035-003; Jackson ImmunoResearch Laboratories, West Grove, PA) for 1 hr. Finally, the culture plates were washed with PBS and the infected cells visualized after exposure to H_2_O_2_-diaminobenzidine (Sigma, St Louis, MO, USA). The number of foci was counted under a light microscope.

### Mouse model of lethal infection

Mice lacking both type I and type 2 IFN receptors were obtained by crossing IFN-α/βR KO mice (kindly provided by Dr. Ishii, Immunology Frontier Research Center, Osaka University) with IFN-γR KO mice (Jackson Laboratories); the progeny were named IFN-α/β/γR KO mice. IFN-α/β/γR KO mice were bred and maintained under specific pathogen-free conditions. Mice were challenged either intraperitoneally or subcutaneously with 2×10^2^-2×10^6^ FFU of DV3P12/08 under anesthesia (by i.p. injection of medetomidine, midazolam, and butorphanol tartrate at final concentrations of 0.3 mg/kg, 4 mg/kg, and 5 mg/kg respectively). Following inoculation, mice were weighed and visually monitored at least once daily by scoring morbidity. Morbidity scoring criteria were based on a 0 to 4 scale: 0-clinically healthy; 1-mild signs of lethargy; 2-lethargy, ruffled fur and hunched posture; 3-lethalgy, ruffled fur, hunched posture with decreased mobility; 4-moribund with unresponsiveness, and/or difficulty walking. Analgesics and anesthetics were not used in this study to minimize animal suffering or distress. Mice were euthanized for humane purposes if they reached a score of four or exhibited weight loss > 20% of initial body weight in the 3-day period after infection, or 25% of weight loss in the 7-day period after infection to avoid unnecessary suffering. No unexpected death occurred during this study and mice were euthanized by cervical dislocation under anesthesia (by i.p. injection of medetomidine, midazolam, and butorphanol tartrate at final concentrations of 0.3 mg/kg, 4 mg/kg, and 5 mg/kg respectively). Euthanized mice were counted as being dead on the following day for analysis.

### Quantification of viral RNA

Viral RNA was isolated from serum (70 μl) using the QIAmp Viral RNA Mini kit (Qiagen) and from the tissue homogenate using TRIzol reagent (Life Technologies) according to the manufacturer’s protocol. Spleen, liver, kidney, thymus, lung, brain, PEC, bone marrow, small intestine, and large intestine were homogenized using a bead crusher μT-12 (Taitec). Total RNA was extracted using TRIzol and adjusted to 200 μg/ml for use in real-time PCR. RNA was quantified using a One-Step SYBR PrimeScript RT-PCR Kit II (Takara) and the following dengue group-specific primers: DN-F, 5’-CAATATGCTGAAACGCGAGAGAAA-3’ and -DN-R, 5’-CCCCATCTATTCAGAATCCCTGCT-3’ [8]. The reaction conditions were as follows: 50°C for 30 min, 95°C for 15 min, and then 40 cycles of 95°C for 20 sec, 55°C for 30 sec, and 72°C for 30 sec, followed by a melting curve analysis step. PCR was performed in a CFX Real-Time PCR Detection System (Bio-Rad).

### Quantitation of vascular permeability

Vascular leakage was examined by intravascular administration of Evans blue (Sigma-Aldrich) as previously described [9]. Briefly, Evans blue (0.2 ml of a 0.5% solution in PBS) was intravenously injected into moribund mice on Day 5–6 post-infection (p.i.) with DV3P12/08. After 2 h, the mice were anesthetized (by i.p. injection of medetomidine, midazolam, and butorphanol tartrate at final concentrations of 0.3 mg/kg, 4 mg/kg, and 5 mg/kg respectively), euthanized by collection of whole blood, extensively perfused with PBS, and the spleen, liver, kidney, lung, brain, small intestine, and large intestine were collected. Evans blue was extracted from the organs by incubation in 1 ml of formamide (Sigma-Aldrich) for 24 hrs, centrifuged at 5,000 rpm for 10 min, and 150 ml of supernatant was collected. The concentration of Evans blue in each organ was quantified by measuring the absorbance at 620 nm using a Corona Grating Microplate Reader SH-9000 (Corona, Electric Co., Ltd.). The results were expressed as optical density per gram of tissue weight.

### Histology

Mice were euthanized, and tissues were harvested and immediately fixed in 10% formalin in PBS. Fixed tissues were paraffin embedded, sectioned and stained with Hematoxylin and Eosin (H&E).

### Measurement of aspartate aminotransferase (AST) and alanine aminotransferase (ALT) levels in mouse serum

Serum was obtained from blood samples on Day 6 p.i. and the levels of AST and ALT measured immediately using Transaminase CII-Test Wako (Wako Chemicals) according to the manufacturer’s instructions. The OD was measured at 555 nm in a Corona Grating Microplate Reader SH-9000 (Corona, Electric Co., Ltd.). The OD values were converted to Karmen units by using AST and ALT calibration curves.

### Cytometric Bead Assay (CBA)

The levels of TNF-α, IL-6, IL-10, MCP-1, IFN-γ, and IL-12p70 in blood were measured using a Mouse Inflammation CBA Kit (Becton Dickinson). Briefly, sera were diluted 10-fold in Assay Diluent and 50 μl of diluted sample was incubated with 50 μl of mixed Mouse Inflammation Capture Beads and 50 μl of Mouse Inflammation PE Detection Reagent for 2 hrs. After washing the mixture once in Wash Buffer, the intensities of the signals on beads were measured by flow cytometry, and the results were analyzed using FCAP Array software 3.0 (BD). All steps were performed at room temperature. Cytokine concentrations were expressed as pg/ml.

### Neutralization of TNF-α and IL-6, and Angiopoietin-1 (Ang-1) treatment

Mice were intraperitoneally injected with 100 μg of a purified, functional grade anti-mouse TNF-α antibody (Ab) (clone MP6-XT3; eBioscience), with 500 μg of anti-mouse IL-6 Ab (MP5-20F3; BioXcell), or with 100 μg of an isotype control Ab (clone P3.6.2.8.1; eBioscience) on Days 1, 2, and 4 p.i. Mice were monitored daily until Day 30 p.i. For treatment with Ang-1, mice were intraperitoneally injected with 1μg of rhAng-1 (R&D Systems)(dissolved in 100 μL phosphate-buffered saline per injection) or the same volume of control buffer (100 μL phosphate-buffered saline per injection) [10].

### Measurement of TNF-α and IL-6 mRNA

The levels of TNF-α and IL-6 mRNA were determined by real-time PCR [11] using the CFX Real-Time PCR Detection System (Bio-Rad). mRNA expression was quantified using the comparative Ct method [12] and levels were normalized to that of GAPDH mRNA. mRNA expression in IFN-α/β/γR KO mice was calculated relative to that in mock (PBS)-inoculated IFN-α/β/γR KO mice.

### Blood test

Heparin blood samples (20–30 μL) were collected from mouse tails on 6 day p.i. Platelet counts were measured by Celltac α MKE-6308 (NIHON KOHDEN, Tokyo, Japan).

### Data analysis

All data were analyzed using Graphpad Prism software (Graphpad 5, San Diego, CA). Kaplan-Meier survival curves were analyzed by the log rank test. Differences in vascular leakage, cytokine levels, and AST and ALT levels were analyzed by Student t-test.

## Results

### DV3P12/08 causes a fatal non-neurological disease in mice

**Fig 1:**
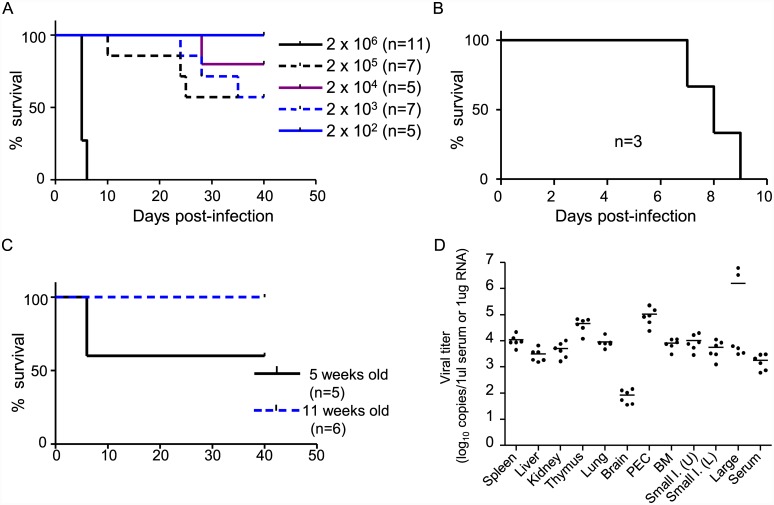
Survival rates and virus titers in IFN-α/β/γR KO mice infected with DV3P12/08. (A) Groups of IFN-α/β/γR KO mice (5–6 weeks old) were intraperitoneally infected with DV3P12/08 at doses ranging from 2 × 10^6^ to 2 × 10^2^ focus-forming units (FFU) or (B) subcutaneously infected with 2.6 × 10^6^ FFU of DV3P12/08 and survival monitored. (C) Groups of IFN-α/βR KO mice (either 5 weeks old or 11 weeks old) were intraperitoneally infected with 1.3 × 10^7^ FFU of DV3P12/08 and survival was monitored. (D) IFN-α/β/γR KO mice (5–6 weeks old) were intraperitoneally infected with 2 × 10^6^ of DV3P12/08, sacrificed at Day 5 p.i. under anesthesia, and perfused extensively with PBS. Virus copy numbers in the spleen, liver, kidney, thymus, lung, brain, peritoneal exudate cells (PEC), bone marrow (BM), upper (U) small intestine, lower (L) small intestine, large intestine, and serum were then measured by qRT-PCR. Bars indicate the mean value. Each symbol represents an individual mouse.

We first examined different DENV strains in IFN-α/β/γR KO mice to identify a strain with high virulence (Table 1). A single DENV-3 strain, DV3P12/08 caused a lethal infection in IFN-α/β/γR KO mice. To further test the virulence of DV3P12/08, IFN-α/β/γR KO mice (5–6 weeks old) were inoculated (i.p.) with viral doses ranging from 2 × 10^6^ to 2 × 10^2^ FFU (Fig 1A). The results showed that infection with 2 × 10^6^ FFU DV3P12/08 induced 100% mortality by Day 6 p.i; thus the infection progressed rapidly. Infection with 2 × 10^5^ FFU resulted in a survival rate of 66%. There was a reciprocal correlation between viral dose and time of death in mice infected with a lethal dose of virus. Although mice showed no symptoms during the initial stages post-infection, they adopted a hunched posture with ruffled fur and suffered severe diarrhea for 1 or 2 days prior to death. Subcutaneous (s.c) infection with 2.6 × 10^6^ FFU DV3P12/08 resulted in death between Days 7 and 9 p.i. (Fig 1B). Mice showed no clinical signs of neurological disorders, such as paralysis, at any time.

**Table 1:** Challenge of DENV clinical isolates in IFN-α/β/γR KO mice.

Virus	Titer (FFU)	Number ^a^	% Survival	Period ^b^
**DV3-2**	3.3×10^5^	4	100	-^c^
**DV3-3**	3.1×10^5^	5	100	-^c^
**DV3-4**	8.3×10^5^	5	40	25–32
**P12/08**	2.0×10^5^	3	0	9–24

^a^ Numbers of mice used for challenge.

^b^ Period indicates the days when mice condition reached the end point.

^c^ All mice survived until days 42 post-infection without symptom.

We next examined the virulence of DV3P12/08 in mice lacking only type I IFN receptors (IFN-α/βR KO mice). Two groups of IFN-α/βR KO mice (either 5 weeks or 11 weeks old) were challenged with 1.3 × 10^7^ FFU of DV3P12/08. All of the 11-week-old mice and 60% of the 5-week-old mice survived (Fig 1C). The surviving 5-week-old mice showed some clinical signs of disease, such as a hunched posture and ruffled fur, at Days 5 and 6 p.i.; however, they subsequently recovered. Therefore, we used IFN-α/β/γR KO mice for all subsequent experiments because they were 100% susceptible to lethal infection with DV3P12/08.

To better understand the disease caused by DV3P12/08, we examined the spleen, liver, kidneys, thymus, lungs, brain, PEC, BM, upper and lower small intestine, large intestine, and serum samples to determine the tissue tropism of the virus. We detected high levels of virus in these organs at Day 5 p.i. (the exception was the brain [< 10^2^ copies/μg RNA) (Fig 1D). Particularly high levels were detected in the large intestine (> 10^6^ copies/μg RNA), thymus, and PEC (> 10^5^ copies/μg RNA).

### DV3P12/08 induces vascular permeability in infected mice

**Fig 2:**
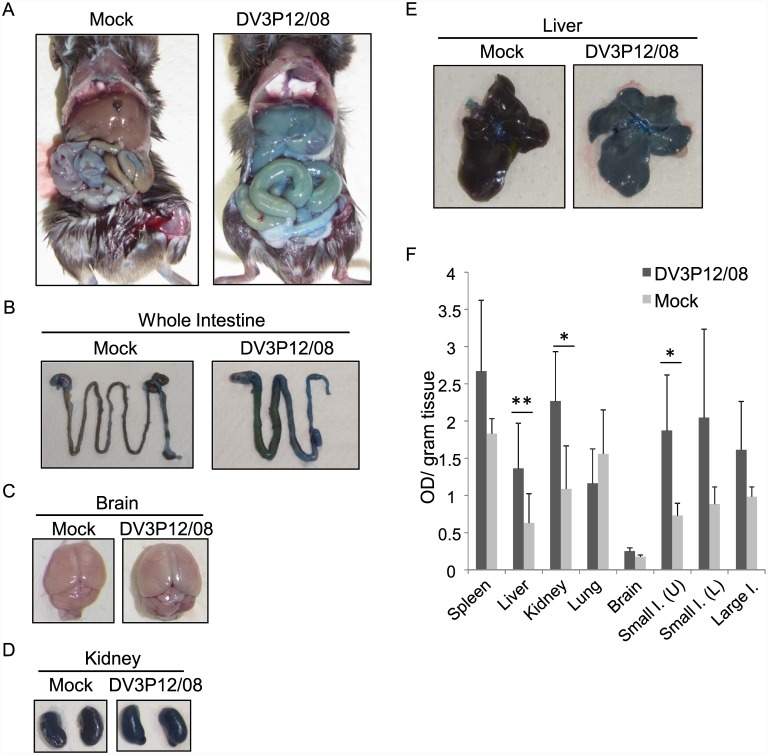
IFN-α/β/γR KO mice infected with DV3P12/08 suffer vascular leakage. (A) Mice (5–6 weeks old) were intraperitoneally infected with 2 × 10^6^ focus-forming units (FFU) of DV3P12/08 or PBS (mock-infected). Moribund mice were intravenously injected with Evans blue dye on Day 5 p.i., sacrificed under anesthesia, and perfused extensively with PBS. Extravasation of the dye into the peritoneal cavity, (B) intestines, (C) brain, (D) kidneys, and (E) liver of mock-infected or DV3P12/08-infected mice. (F) Quantification of Evans blue in the spleen, liver, kidneys, lungs, brain, upper small intestine, lower small intestine, and large intestine of infected and mock-infected mice (n = 6/group). *P < 0.05 and **P < 0.01.

The rapidly progressing fatal non-neurologic disease observed in DV3P12/08-infected IFN-α/β/γR KO mice suggested that DV3P12/08 may cause increased vascular permeability, a hallmark of severe DENV infection. Therefore, we examined vascular leakage by injecting DV3P12/08-infected or mock-infected mice with Evans blue dye [6, 9]. We observed leakage of Evans blue into the liver, small intestine, and kidneys of DV3P12/08-challenged mice at Day 5 p.i. (the moribund stage) (Fig 2A–2E). We next extracted the dye from the organs to measure the amount of leakage. The highest levels of vascular leakage occurred in the liver, kidney, and small intestine (Fig 2F). These observations suggest that DV3P12/08 causes increased vascular permeability in certain tissues.

### Histological examination of organs harvested from DV3P12/08-infected mice

**Fig 3:**
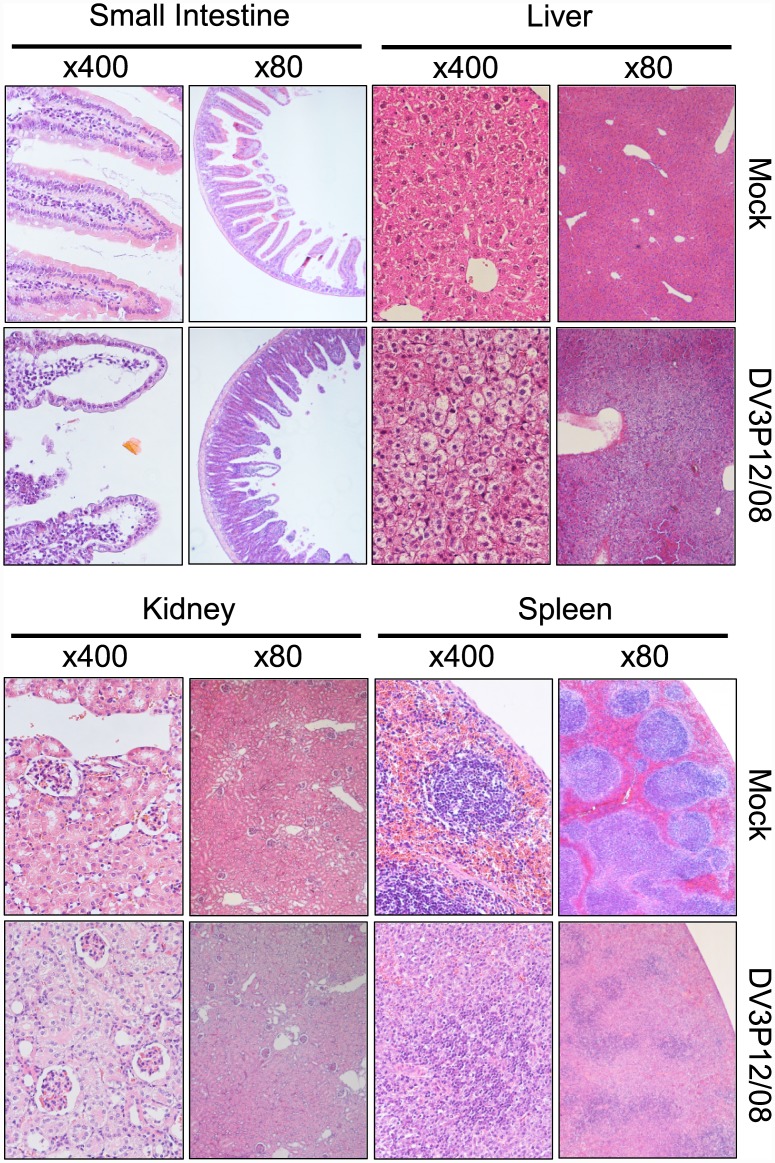
Histopathological examination of tissues from IFNα/β/γR KO mice infected with DV3P12/08. IFN-α/β/γR KO mice infected with 2 × 10^6^ focus-forming units (FFU) of DV3P12/08 or mock-infected with PBS were euthanized at Day 6 p.i. Sections of liver, spleen, kidney, and small intestine were prepared, stained with hematoxylin and eosin, and observed under low (×100) and high (×400) magnification. Images are representative of at least three sections per tissue.

We next performed histologic analysis of hematoxylin and eosin-stained tissue sections from the organs of IFN-α/β/γR KO and control mice. The livers from mice infected with 2 × 10^6^ FFU of DV3P12/08 showed evidence of an inflammatory infiltrate, edema, and swollen hepatocytes (suggestive of hepatocyte injury) (Fig 3); however, there was no evidence of necrosis. Although mice infected with 2 × 10^6^ FFU of DV3P12/08 suffered severe diarrhea and exhibited marked vascular leakage in the intestine (Fig 2F), the edematous changes and vascular dilation in this organ were not severe; however, we did observe mild-to-moderate edema and increased numbers of infiltrating cells in the lamina propria. Also, epithelial cells were detached from the tops of the villi. These features provide evidence for pathological changes in tissues exhibiting signs of increased vascular permeability. In addition, a reduction in the length of the intestine was observed (Fig 2B), suggesting the intestine had undergone a strong inflammatory response. Massive loss of lymphocytes from the white pulp and infiltration of inflammatory leukocytes were observed in the spleen at Day 5 p.i. Although there was evidence of vascular leakage in the kidney (Fig 2F), there were no clear pathological changes. The glomeruli and tubules appeared normal.

### Cytokine levels in DV3P12/08-infected mice

**Fig 4:**
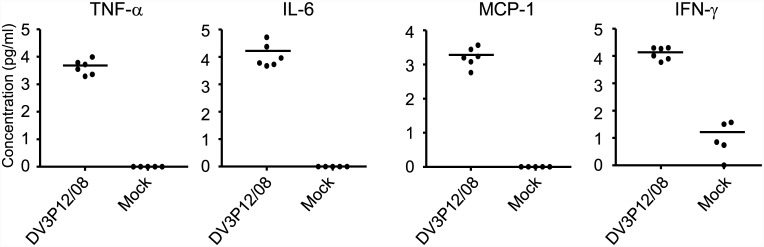
Levels of pro-inflammatory cytokines and chemokines in DV3P12/08-infected IFN-α/β/γR KO mice. Mice were intraperitoneally infected with 2 × 10^6^ focus-forming units (FFU) of DV3P12/08 or PBS (mock) and blood samples taken at Day 6 p.i. The levels of TNF-α, IL-6, MCP-1, IFN γ, and IL-10 were then measured using a Mouse Inflammation CBA Kit. The results are expressed as the mean + SD of 5–6 mice per group.

Cytokines are thought to contribute to the vascular leakage associated with severe dengue [5, 13, 14]. Therefore, we measured the levels of five pro-inflammatory cytokines (TNF-α, IL-6, IL-10, IL-12p70, IFN-γ) and one chemokine (MCP-1) in serum samples collected from mice (infected with 2 × 10^6^ FFU of DV3P12/08) at Day 5 p.i. The results showed that the levels of TNF-α, IL-6, IFN-γ, and MCP-1 were much higher in infected mice than in mock-infected mice (Fig 4). No IL-12p70 or IL-10 were detected (data not shown). These data suggest that infected mice experience a cytokine storm, which results in severe vascular leakage.

### Neutralizing TNF-α prevents DV3P12/08-induced lethality during the acute phase of disease

**Fig 5:**
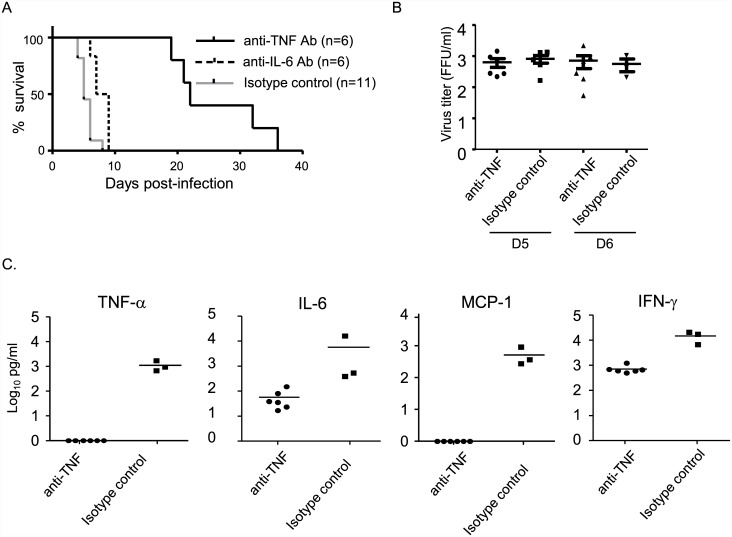
TNF-α plays a critical role in lethal DV3 12/08 infection. (A) Survival of IFN-α/β/γR knockout (KO) mice intraperitoneally infected with 2 × 10^6^ focus-forming units (FFU) of DV3P12/08, followed by the intraperitoneal injection with an anti-TNF-α Ab (n = 6) or an isotype control Ab (n = 6) on Days 1, 2, 4 p.i. Survival differences were statistically significant between the anti-TNF-α-treated group and the isotype control (*p = 0*.*0004*), the anti-TNF-α-treated group and the anti-IL6-treated group (*p = 0*.*0023*), the anti-IL6-treated group and the isotype control (*p = 0*.*0036*). (B) Sera were collected from anti-TNF-α Ab- (n = 6) or isotype control Ab- (n = 5) treated mice on Days 5 and 6 p.i. and the viral copy number determined by qRT-PCR. (C) Sera were collected from mock- (n = 3) or DV3P12/08 (2 × 10^6^ FFU) (n = 6) infected mice and the levels of TNF-α, IL-6, MCP-1, IFN-γ were measured by qRT-PCR. Bars indicate the mean values. Each symbol represents an individual mouse.

To examine whether TNF-α or IL-6 is responsible for the lethal effects of the virus in this animal model, we treated DV3P12/08-infected IFN-α/β/γR KO mice with either an anti-mouse TNF-α Ab (100 μg), anti-IL-6 antibody (500 μg) or an isotype control antibody (100 μg) on Days 1, 2 and 4 p.i. Treatment with the anti-TNF-α Ab significantly prolonged the survival of DV3P12/08-infected mice until Day 18–36 p.i. (Fig 5A); however, most of the control mice died by Day 5–7 p.i. On the other hand, treatment with the anti-IL-6 Ab weakly protected mice (Fig 5A). This result clearly shows that TNF-α plays a critical role in the lethal effects of DENV in this IFN-α/β/γR KO mouse model. To examine whether anti-TNF-α treatment had any effect on virus production, we measured the viral titers in the sera of mice treated (or not) with the anti-TNF-α antibody. TNF-α treatment had no effect on virus production at Days 4 or 5 p.i. (Fig 5B); thus neutralization of TNF-α protected mice from lethal infection without affecting virus production, suggesting that TNF-α plays a key role in lethal infection.

### Neutralizing TNF-α suppresses IL-6, IFN-γ, and MCP-1 levels

We next examined whether neutralizing of TNF-α affected the levels of other cytokines. Serum samples were collected from DV3P12/08-infected mice treated with or without anti-TNF-α Ab on Day 5 p.i. and the levels of TNF-α, IL-6, IL-10, IL-12p70, IFN-γ and MCP-1 were measured. As expected, treatment with the anti-TNF-α Ab abolished TNF-α; however, we also found that it markedly suppressed the levels of IL-6 and MCP-1 (Fig 5C). This suggests that increased expression of TNF-α has an effect on the levels of these two molecules. It is important to note, however, that the protective effects of the anti-TNF-α Ab may depend on factors other than (or in addition to) TNF-α.

### Neutralization of TNF-α relieves liver damage

**Fig 6:**
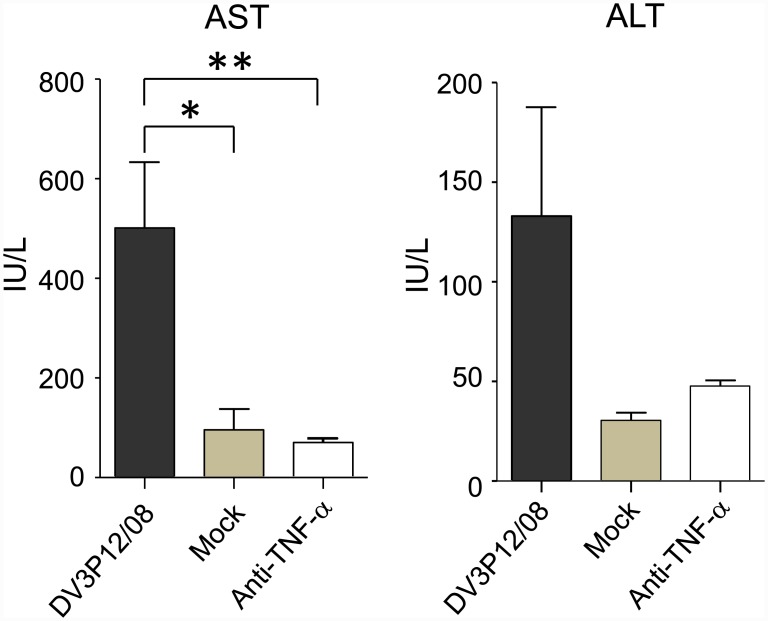
Serum alanine (AST) and aspartate (ALT) transaminase levels in DV3P12/08-infected IFN-α/β/γR KO mice. Mice were intraperitoneally infected with 2 × 10^6^ focus-forming units (FFU) of DV3P12/08 or with PBS (mock) and blood samples were taken at Day 5 p.i. The levels of AST and ALT were measured using Transaminase CII-Test Wako. The results are expressed as the mean + SD of 5–6 mice per group. *P < 0.05 and **P < 0.01.

Obvious vascular leakage (Fig 2) and pathological change (Fig 3) were observed in liver. Liver damage was evaluated by measuring AST and ALT levels in serum collected from IFN-α/β/γR KO mice at Day 6 p.i. Levels of AST and ALT were significantly increased in DV3P12/08-infected mice, and importantly there was no increase in AST and ALT levels in anti-TNF-α Ab treated-DV3P12/08-infected mice (Fig 6). This suggests that liver damage was triggered by the induction of TNF-α.

### Expression of TNF-α and IL-6 mRNA in different organs

**Fig 7:**
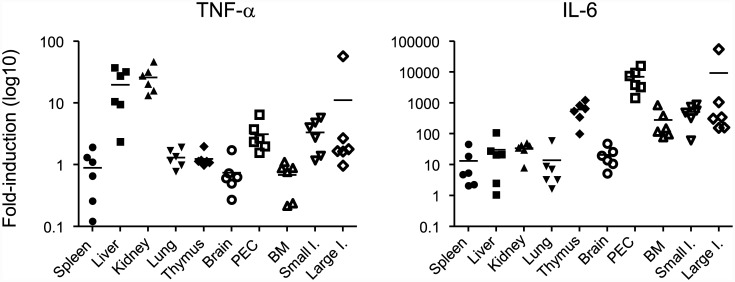
Expression of TNF-α and IL-6 mRNA in organs from IFN-α/β/γR KO mice. Mice were infected with PBS (n = 3) or with 2 × 10^6^ focus-forming units (FFU) of DV3P12/08 (n = 6) and total RNA was extracted from the spleen, liver, kidneys, lungs, thymus, brain, peritoneal exudate cells (PEC), bone marrow (BM), small intestine, and large intestine at Days 6 p.i., and the levels of TNF-α and IL-6 mRNA were measured by qRT-PCR. The results were analyzed using the comparative Ct method. Dots represent individual mice and bars represent the mean values.

Vascular leakage was mainly evident in three organs: liver, kidney, and small intestine (little leakage was observed in the brain) (Fig 2F). Vascular leakage appeared to be a local event, even though cytokine levels in the serum were elevated (Fig 5). This raised the following question: which organ is responsible for the production of TNF-α and IL-6? To answer this, we examined the expression of TNF-α and IL-6 mRNA in different organs by quantitative real-time PCR. We found that high levels of TNF-α mRNA were expressed in the liver and kidney (19.7- and 25.8-fold increase relative to that in mock-infected control, respectively) (Fig 7). Although vascular leakage was observed in the small intestine, levels of TNF-α was low. On the other hand, high levels of IL-6 mRNA were detected in the thymus, PEC, BM, small intestine, and large intestine (616-, 6,934-, 279-, 512-, and 9,396-fold, respectively). On the other hand, much lower levels are expressed in the liver and kidney (30- and 34-fold, respectively).

### Angiopoietin-1 (Ang-1) protects IFN-α/β/γR KO mice

**Fig 8:**
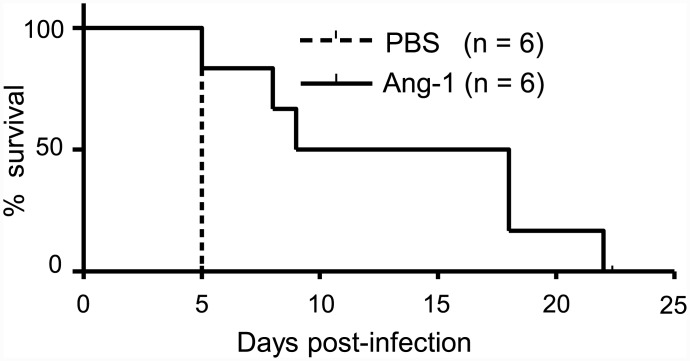
Ang-1 protects DV3P12/08-infected IFN-α/β/γR KO mice. Survival of IFN-α/β/γR knockout (KO) mice intraperitoneally infected with 2 × 10^6^ focus-forming units (FFU) of DV3P12/08, followed by the intraperitoneal injection with Ang-1 (1 μg/mouse/time) (n = 6) or PBS control Ab (n = 6) on Days 1, 3, 5 p.i. Survival differences were statistically significant between the Ang-1 and PBS-treated mice (*p = 0*.*0051*).

Recently, mRNA expression of endothelial tyrosine kinases Tie1 and Tie2 was found to be reduced in susceptible mice infected with Ebola virus [15]. In this mouse model, hemorrhagic syndrome was observed. Ang-1 is a ligand of the Tie2 receptor [16] and acts to prevent vascular leakage [17]. To examine whether introduction of Ang-1 protects IFN-α/β/γR KO mice from lethal infection with P12/08, mice were treated with recombinant rhAng-1 at Days 1, 3, and 5 p.i. With no treatment (Fig 8), all mice died at Day 5 while with Ang-1 treatment, 50% of mice survived until Day 17 p.i. and 10% of mice survived until day 21 p.i. This suggests that the Tie/Angiopoietin system plays a critical role in this model.

### DV3P12/08 does not cause thrombocytopenia

**Fig 9:**
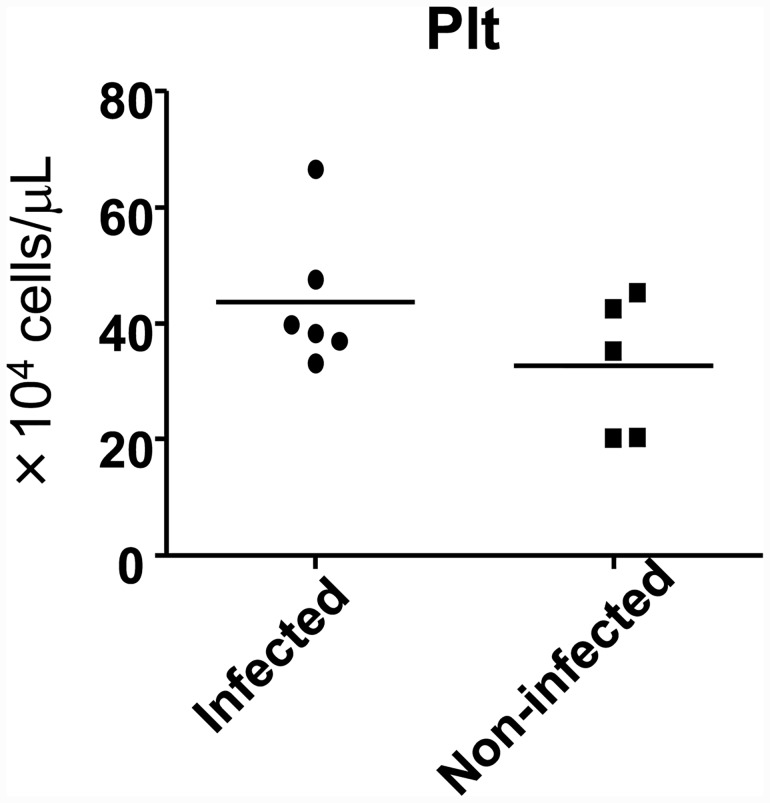
Analysis of blood samples obtained from IFN-α/β/γR KO mice infected with DV3P12/08. Blood was harvested from mice infected with PBS (mock) (n = 5) or 2 × 10^6^ focus-forming units (FFU) of DV3P12/08 (n = 6) at Days 6. p.i and the number of platelets was counted by using Celltac a (Nihon Kohden). *P* = 0.04.

In addition to vascular leakage, another typical manifestation is thrombocytopenia. However, we found no evidence for thrombocytopenia in this model (Fig 9). Mechanisms, other than loss of thrombocytes, may be responsible for the vascular leakage and thrombocytopenia.

## Discussion

Here, we showed that DDV3P12/08, a low passaged recent clinical isolate DV3P12/08 caused acute systemic lethal infection in C57BL/6 background IFN-α/β/γR KO mice. Viral infection caused a DHF-like syndrome, which was associated with vascular leakage and liver damage. Several groups have used AG129 mice infected with a mouse-adapted DENV-2 strain and non-mouse adapted strain as an animal model of DENV infection [6, 18–21]. Unfortunately, the majority of DENV clinical isolates and laboratory strains that we tested did not cause lethal infection in mice, not even in IFN-α/β/γR KO mice. Although some strains did cause a lethal infection, the disease was manifested by neurological disorders rather than vascular leakage (Phanthanawiboon et al. submitted for publication). However, there may be other as-yet-unidentified strains of DENV that cause a DHF-like syndrome, at least in mice lacking IFN receptors. Indeed, Sarathy et al. reported that DENV-3 C0360/94 strain caused systemic lethal infection [22]. There remains an urgent need to identify virulent strains of DENV 1 and 4 because there are currently no mouse models of infection by these serotypes.

The model used in the present study has features that are similar to those of DENV-2 models based on infection with a mouse-adapted DENV strain, D2S10 [6], D2Y98P strains [21], and the DENV-3 C0360/94 [22] strain (in AG129 mice). Mice infected with these viruses suffered, systemic infection and vascular leakage, accompanied by histopathological changes such as dilated hepatocytes in the liver, mononuclear cell infiltration and loss of texture in the spleen, thickening of the mucosal layer of the intestine due to the presence of inflammatory cells, and disease amelioration after treatment with a neutralizing anti-TNF-α Ab [6, 21, 22]. We also noted an apparent lack of fluid in the pleural cavity (Fig 2) and the absence of thrombocytopenia (Fig 9), as was observed in a previous study [6] although the DENV-3 C0360/94 strain caused thrombocytopenia [22]. Despite the fact that the IFN-α/β/γR KO mouce used in this study have the C57BL/6 genetic background, which is different from that of AG129 mice, DV3P12/08 infection caused similar symptoms in this mouse. There are a number of genetic differences between C57BL/6 and AG129 mice; however, these differences do not appear to affect the phenotype of mice infected with DENV.

Although most observations made using this model resemble those made using other models, there are several differences such as the absence of thrombocytopenia, as above mentioned. In addition, swelling of intestine was very obvious (Fig 2A) and intestine length was shorter in this model than other models (Fig 2B). These features of the intestine are often observed in some inflammatory enteric diseases such as inflammatory bowel diseases (IBD) [23]. IBD is thought to be driven by several pro-inflammatory cytokines, especially IL-6 [24]. The change in appearance of the intestine (Fig 7) may be due to the expression of IL-6 in the intestine. Although vascular leakage was observed in the intestine of all models using AG129 mice, virus was not detected in the intestine of mice infected with D2Y98D [21]. In other models, virus was detected in the intestine from mice infected with D2S10 [6] and C0360/94 [22]. In this model, DENV infection in the intestine induced induce vascular leakage, as evidenced by the high levels of DV3P12/08 found in the intestines (Fig 1D).

TNF-α is likely to be an important factor in all mouse models [6, 25, 26]; however, the protective mechanism of anti-TNF-α neutralizing Ab has not been investigated. Neutralization of TNF-α suppressed IL-6, MCP-1, and IFN-γ levels in serum (Fig 5C). Although IL-6 was also partly involved in lethality (Fig 5A), IL-6 was probably induced by TNF-α. These results suggest that there may be other mediators triggered by TNF-α that are responsible for lethality and vascular leakage. Importantly, neutralization of TNF-α relieved liver damage (Fig 6), suggesting a critical role for liver function in the lethality of this model. Interestingly, C0360/94 did not induce liver damage [22]. Both type 3 DENVs results in lethal infection, but the detailed events leading up to lethality may be different at the organ level. A comparative study will be required to address this issue in the future.

TNF-α circulates in serum and is likely to be a master regulator (Fig 4), but vascular leakage was limited mainly to the liver, kidney, and intestine (Fig 2). This raises the following question: What is the determinant of organ specific vascular leakage? Which organs or cells produce TNF-α? The results of the present study suggest that cells within the liver and kidney are the most likely source (Fig 7), which is consistent with the pattern of vascular leakage observed in this model (Fig 2F). However, a question remains. Although marked vascular leakage was observed in the intestine (Fig 2), we observed high levels of IL-6 mRNA instead of the low levels of TNF-α mRNA (Fig 7); thus, vascular leakage in this organ might be triggered by IL-6 produced in the intestine. We propose that vascular leakage in different organs may be triggered by different mechanisms.

A significant advance in this study was the observation that Ang-1 effectively extended the mouse survival (Fig 8). Ang-1 is a ligand for the endothelial specific receptor, tyrosine kinase with Ig-like loops and epidermal growth factor homology domains-2 (Tie2) [16] and is essential for embryonic vascular development [27]. In addition, Thurston et al. reported that Ang-1 protects the adult vasculature against vascular leakage [17]. Binding of Ang-1 to Tie2 receptor activates Tie2, and angiopoietin-2 (Ang-2) is an antagonist of Ang-1. It has been believed that Ang-2 is thought to inactivate Tie2 and destabilize vessels by dissociating endothelial cells into endothelial cells and endothelial cells into mural cells [28]. The Ang-1/Ang-2 balance is one of determinants governing the stability of blood vessels. Ang-1 mediated protection against vascular leakage caused by several diseases has been reported. Vessels in Ang-1 overexpressing mice are resistant to leakage caused by inflammatory agents [29]. Moreover, Witzenbichler et al. reported a protective role for Ang-1 in endotoxin shock [30], which is a condition with microvascular leakage. Importantly, a lower Ang-1/Ang-2 ratio was found in dengue patients with severe condition [31, 32]. In addition, an imbalance in the Ang-1/Ang-2 ratio was also observed in patients with Crimean-Congo hemorrhagic fever [33]. Tie 2/Ang signaling may play an important role in other viral hemorrhagic fevers. Interestingly, in human, platelets store Ang-1 [34], suggesting that thrombocytopenia may exacerbate the imbalance in the Ang-1/Ang-2 ratio and lead to condition that cause vascular leakage. The expression patterns of Tie2/Ang were associated with organ-specific vascular leakage (Fig 2). Tie2 and its ligands Ang-1/Ang-2 are highly expressed in the lung of mice [28], [35], which may insensate the lung to activation of Tie2 driven by DENV-infection. A more detailed study is needed to clarify whether the improvement in mouse survival brought about by administration of Ang-1 is due to its indirect blocking effect against a yet unidentified factor that induces vascular leakage or its protective role in the blood vessel restoration step. We still cannot exclude the possible involvement of other factors in vascular leakage, and the nature of the facto(s) that link between TNF-α to Ang-1 is unclear. It will be necessary to determine how the Tie2/Ang system is involved in severe dengue, and answer if we are to develop therapeutics that effectively prevent the vascular leakage observed in DENV-infected patients.

## References

[1] Guzman MG, Halstead SB, Artsob H, Buchy P, Farrar J, Gubler DJ, et al Dengue: a continuing global threat. Nature reviews Microbiology. 2010;8(12 Suppl):S7–16. Epub 2010/11/17. 10.1038/nrmicro2460 21079655 PMC4333201

[2] Whitehorn J, Simmons CP. The pathogenesis of dengue. Vaccine. 2011;29(42):7221–8. Epub 2011/07/26. 10.1016/j.vaccine.2011.07.022 21781999

[3] Chambers TJ, Hahn CS, Galler R, Rice CM. Flavivirus genome organization, expression, and replication. Annual review of microbiology. 1990;44:649–88. Epub 1990/01/01. 10.1146/annurev.mi.44.100190.003245 2174669

[4] Srichaikul T, Nimmannitya S. Haematology in dengue and dengue haemorrhagic fever. Bailliere's best practice & research Clinical haematology. 2000;13(2):261–76. Epub 2000/08/16. 10.1053/beha.2000.0073 10942625

[5] Kurane I. Dengue hemorrhagic fever with special emphasis on immunopathogenesis. Comparative immunology, microbiology and infectious diseases. 2007;30(5–6):329–40. Epub 2007/07/25. 10.1016/j.cimid.2007.05.010 17645944

[6] Shresta S, Sharar KL, Prigozhin DM, Beatty PR, Harris E. Murine model for dengue virus-induced lethal disease with increased vascular permeability. Journal of virology. 2006;80(20):10208–17. 10.1128/JVI.00062-06 17005698 PMC1617308

[7] Setthapramote C, Sasaki T, Puiprom O, Limkittikul K, Pitaksajjakul P, Pipattanaboon C, et al Human monoclonal antibodies to neutralize all dengue virus serotypes using lymphocytes from patients at acute phase of the secondary infection. Biochemical and biophysical research communications. 2012;423(4):867–72. 10.1016/j.bbrc.2012.06.057 22713454

[8] Shu PY, Chang SF, Kuo YC, Yueh YY, Chien LJ, Sue CL, et al Development of group- and serotype-specific one-step SYBR green I-based real-time reverse transcription-PCR assay for dengue virus. Journal of clinical microbiology. 2003;41(6):2408–16. Epub 2003/06/07. 12791857 10.1128/JCM.41.6.2408-2416.2003 PMC156548

[9] Thompson LF, Eltzschig HK, Ibla JC, Van De Wiele CJ, Resta R, Morote-Garcia JC, et al Crucial role for ecto-5'-nucleotidase (CD73) in vascular leakage during hypoxia. The Journal of experimental medicine. 2004;200(11):1395–405. Epub 2004/12/08. 10.1084/jem.20040915 15583013 PMC1237012

[10] David S, Park JK, Meurs M, Zijlstra JG, Koenecke C, Schrimpf C, et al Acute administration of recombinant Angiopoietin-1 ameliorates multiple-organ dysfunction syndrome and improves survival in murine sepsis. Cytokine. 2011;55(2):251–9. 10.1016/j.cyto.2011.04.005 21531574

[11] Overbergh L, Giulietti A, Valckx D, Decallonne R, Bouillon R, Mathieu C. The use of real-time reverse transcriptase PCR for the quantification of cytokine gene expression. Journal of biomolecular techniques: JBT. 2003;14(1):33–43. Epub 2003/08/07. 12901609 PMC2279895

[12] Schmittgen TD, Livak KJ. Analyzing real-time PCR data by the comparative C(T) method. Nature protocols. 2008;3(6):1101–8. Epub 2008/06/13. 18546601 10.1038/nprot.2008.73

[13] Lei HY, Yeh TM, Liu HS, Lin YS, Chen SH, Liu CC. Immunopathogenesis of dengue virus infection. Journal of biomedical science. 2001;8(5):377–88. Epub 2001/09/11. 11549879 10.1007/BF02255946

[14] Rothman AL. Immunity to dengue virus: a tale of original antigenic sin and tropical cytokine storms. Nature reviews Immunology. 2011;11(8):532–43. 10.1038/nri3014 21760609

[15] Rasmussen AL, Okumura A, Ferris MT, Green R, Feldmann F, Kelly SM, et al Host genetic diversity enables Ebola hemorrhagic fever pathogenesis and resistance. Science. 2014;346(6212):987–91. 10.1126/science.1259595 25359852 PMC4241145

[16] Davis S, Aldrich TH, Jones PF, Acheson A, Compton DL, Jain V, et al Isolation of angiopoietin-1, a ligand for the TIE2 receptor, by secretion-trap expression cloning. Cell. 1996;87(7):1161–9. 8980223 10.1016/s0092-8674(00)81812-7

[17] Thurston G, Rudge JS, Ioffe E, Zhou H, Ross L, Croll SD, et al Angiopoietin-1 protects the adult vasculature against plasma leakage. Nature medicine. 2000;6(4):460–3. 10.1038/74725 10742156

[18] Stein DA, Huang CY, Silengo S, Amantana A, Crumley S, Blouch RE, et al Treatment of AG129 mice with antisense morpholino oligomers increases survival time following challenge with dengue 2 virus. The Journal of antimicrobial chemotherapy. 2008;62(3):555–65. Epub 2008/06/24. 10.1093/jac/dkn221 18567576 PMC7109848

[19] Watanabe S, Rathore AP, Sung C, Lu F, Khoo YM, Connolly J, et al Dose- and schedule-dependent protective efficacy of celgosivir in a lethal mouse model for dengue virus infection informs dosing regimen for a proof of concept clinical trial. Antiviral research. 2012;96(1):32–5. Epub 2012/08/08. 10.1016/j.antiviral.2012.07.008 22867971

[20] Fuchs J, Chu H, O'Day P, Pyles R, Bourne N, Das SC, et al Investigating the efficacy of monovalent and tetravalent dengue vaccine formulations against DENV-4 challenge in AG129 mice. Vaccine. 2014 Epub 2014/09/23. 10.1016/j.vaccine.2014.08.087 PMC4252871 25239488

[21] Tan GK, Ng JK, Trasti SL, Schul W, Yip G, Alonso S. A non mouse-adapted dengue virus strain as a new model of severe dengue infection in AG129 mice. PLoS neglected tropical diseases. 2010;4(4):e672 10.1371/journal.pntd.0000672 20436920 PMC2860513

[22] Sarathy VV, White M, Li L, Gorder SR, Pyles RB, Campbell GA, et al A Lethal Murine Infection Model for Dengue Virus 3 in AG129 Mice Deficient in Type I and II Interferon Receptors Leads to Systemic Disease. Journal of virology. 2015;89(2):1254–66. 10.1128/JVI.01320-14 25392217 PMC4300670

[23] Chassaing B, Aitken JD, Malleshappa M, Vijay-Kumar M. Dextran sulfate sodium (DSS)-induced colitis in mice. Curr Protoc Immunol. 2014;104:Unit 15 25. 10.1002/0471142735.im1525s104 PMC3980572 24510619

[24] Mudter J, Neurath MF. Il-6 signaling in inflammatory bowel disease: pathophysiological role and clinical relevance. Inflamm Bowel Dis. 2007;13(8):1016–23. 17476678 10.1002/ibd.20148

[25] Atrasheuskaya A, Petzelbauer P, Fredeking TM, Ignatyev G. Anti-TNF antibody treatment reduces mortality in experimental dengue virus infection. FEMS immunology and medical microbiology. 2003;35(1):33–42. Epub 2003/02/19. 12589955 10.1111/j.1574-695X.2003.tb00646.x

[26] Ng JK, Zhang SL, Tan HC, Yan B, Maria Martinez Gomez J, Tan WY, et al First experimental in vivo model of enhanced dengue disease severity through maternally acquired heterotypic dengue antibodies. PLoS pathogens. 2014;10(4):e1004031 Epub 2014/04/05. 10.1371/journal.ppat.1004031 24699622 PMC3974839

[27] Gale NW, Yancopoulos GD. Growth factors acting via endothelial cell-specific receptor tyrosine kinases: VEGFs, angiopoietins, and ephrins in vascular development. Genes Dev. 1999;13(9):1055–66. 10323857 10.1101/gad.13.9.1055

[28] Milam KE, Parikh SM. The angiopoietin-Tie2 signaling axis in the vascular leakage of systemic inflammation. Tissue barriers. 2015;3(1–2):e957508 10.4161/21688362.2014.957508 25838975 PMC4372013

[29] Thurston G, Suri C, Smith K, McClain J, Sato TN, Yancopoulos GD, et al Leakage-resistant blood vessels in mice transgenically overexpressing angiopoietin-1. Science. 1999;286(5449):2511–4. 10617467 10.1126/science.286.5449.2511

[30] Witzenbichler B, Westermann D, Knueppel S, Schultheiss HP, Tschope C. Protective role of angiopoietin-1 in endotoxic shock. Circulation. 2005;111(1):97–105. 10.1161/01.CIR.0000151287.08202.8E 15611372

[31] Michels M, van der Ven AJ, Djamiatun K, Fijnheer R, de Groot PG, Griffioen AW, et al Imbalance of angiopoietin-1 and angiopoetin-2 in severe dengue and relationship with thrombocytopenia, endothelial activation, and vascular stability. The American journal of tropical medicine and hygiene. 2012;87(5):943–6. 10.4269/ajtmh.2012.12-0020 22949515 PMC3516273

[32] van de Weg CA, Pannuti CS, van den Ham HJ, de Araujo ES, Boas LS, Felix AC, et al Serum angiopoietin-2 and soluble VEGF receptor 2 are surrogate markers for plasma leakage in patients with acute dengue virus infection. Journal of clinical virology: the official publication of the Pan American Society for Clinical Virology. 2014;60(4):328–35. 10.1016/j.jcv.2014.05.001 24928471

[33] Sancakdar E, Guven AS, Uysal EB, Deveci K, Gulturk E. Important of Angiopoietic System in Evaluation of Endothelial Damage in Children with Crimean-Congo Hemorrhagic Fever. Pediatr Infect Dis J. 2015;34(8):e200–5. 25831422 10.1097/INF.0000000000000706

[34] Huang YQ, Li JJ, Karpatkin S. Identification of a family of alternatively spliced mRNA species of angiopoietin-1. Blood. 2000;95(6):1993–9. 10706866

[35] Parikh SM, Mammoto T, Schultz A, Yuan HT, Christiani D, Karumanchi SA, et al Excess circulating angiopoietin-2 may contribute to pulmonary vascular leak in sepsis in humans. PLoS medicine. 2006;3(3):e46 10.1371/journal.pmed.0030046 16417407 PMC1334221

